# Purification and characterization of recombinant neuraminidase as a potentially broadly protective influenza virus vaccine candidate^☆^

**DOI:** 10.1016/j.vaccine.2025.127471

**Published:** 2025-07-11

**Authors:** Fernando De Mathia, Tobias Kargl, Matthias Müller, Irfan Erdem, Brooks Hayes, Eduard Puente-Massaguer, Florian Krammer, Nico Lingg

**Affiliations:** a BOKU University, Institute of Bioprocess Science and Engineering, Department of Biotechnology and Food Science, Vienna, Austria; b Expression Systems, an Advancion Company, Davis, CA, USA; c Department of Microbiology, Icahn School of Medicine at Mount Sinai, New York, NY, USA; d Department of Pathology, Molecular and Cell-Based Medicine, Icahn School of Medicine at Mount Sinai, New York, NY, USA; e Ignaz Semmelweis Institute, Interuniversity Institute for Infection Research, Medical University of Vienna, Vienna, Austria; f Center for Vaccine Research and Pandemic Preparedness (C-VaRPP), Icahn School of Medicine at Mount Sinai, New York, NY, USA; g acib – Austrian Centre of Industrial Biotechnology, Vienna, Austria

**Keywords:** Influenza, Protein subunit vaccine, Downstream processing, Chromatography, Immobilized metal affinity chromatography, Baculovirus expression system

## Abstract

Influenza viruses pose a significant public health threat, causing seasonal epidemics and occasional pandemics with substantial morbidity and mortality worldwide. The development of effective vaccines remains crucial for mitigating the impact of influenza virus infections. The influenza virus possesses two glycoproteins on its surface: hemagglutinin, which is immunodominant, and neuraminidase, which is immunosubdominant. Traditional influenza vaccines primarily target the viral surface glycoprotein hemagglutinin to induce protective immunity. However, the high mutation rate of this protein, particularly in response to selective pressure from immune responses, limits the durability and efficacy of current vaccines. Efficient production of influenza vaccines relies on scalable purification processes to ensure safe and efficacious antigens. Neuraminidase (NA), the second glycoprotein of influenza virus, has recently emerged as target for broadly protective protein subunit vaccine formulations, necessitating development of robust manufacturing and characterization methods for this antigen. Here we present a scalable approach for purifying recombinant NA, expressed in insect cells utilizing the baculovirus expression system. Lean purification processes, based on chromatography and tangential flow filtration, achieve high yield and purity while maintaining the target’s structural integrity and biological activity. The purified product is characterized through a variety of techniques, which confirm its structural and functional properties, and the consistency of those throughout the experiments performed. Scalability from laboratory to manufacturing scale under good manufacturing practice (GMP) ensures reproducibility, thus advancing the development of recombinant NA-based vaccines for comprehensive influenza control.

## 1. Introduction

Influenza viruses are a substantial threat to public health as they can cause seasonal outbreaks and sporadic pandemics that have a high global morbidity and mortality rate [1–4]. To mitigate the consequences of influenza virus infections, development of better and broadly protective vaccines is essential. The influenza virus has two glycoproteins on its surface: the immunodominant hemagglutinin (HA) and the immunosubdominant neuraminidase (NA) [5]. In order to produce protective immunity, traditional influenza vaccines predominantly target the HA. Unfortunately, the durability and efficacy of currently available vaccines are constrained by the high HA mutation rate [6]. NA is the second surface glycoprotein of influenza virus and an enzyme that binds to and cleaves sialosides from glycans on the surface of host cells. It is thought to be important for several stages of the viral live cycles and immunity to NA can be broadly protective [7,8]. However, current influenza virus vaccines do not induce strong and consistent immune responses to NA [9–11]. Supplementation of current inactivated influenza virus vaccines with recombinant correctly folded NA, or an NA stand-alone vaccine, could potentially increase protection and protective breadth against influenza virus [12]. In a recent study, we were able to express a recombinant neuraminidase construct in insect cells, utilizing the baculovirus expression system [13]. The construct, composed of the N1 globular head domain from A/Michigan/45/2015 (H1N1), the measles virus phosphoprotein tetramerization domain; and a hexahistidine purification tag, was called N1-MPP. This vaccine candidate has been shown to induce full protection against lethal viral challenge in animal models and has been selected for clinical development [13,14]. A similar construct based on an N2 NA has also shown promise in pre-clinical testing [15].

The baculovirus expression system is a valuable tool in molecular biology and biotechnology with a high potential for vaccine manufacturing [16]. Baculoviruses, naturally infecting insect cells, have been harnessed for their ability to efficiently produce complex eukaryotic proteins. The system’s advantages lie in its capacity to accommodate large and structurally intricate genes, essential for proteins requiring post-translational modifications and proper folding [17]. Furthermore, this technology is already used to produce recombinant-protein vaccines for influenza virus [18].

**Fig. 1.**
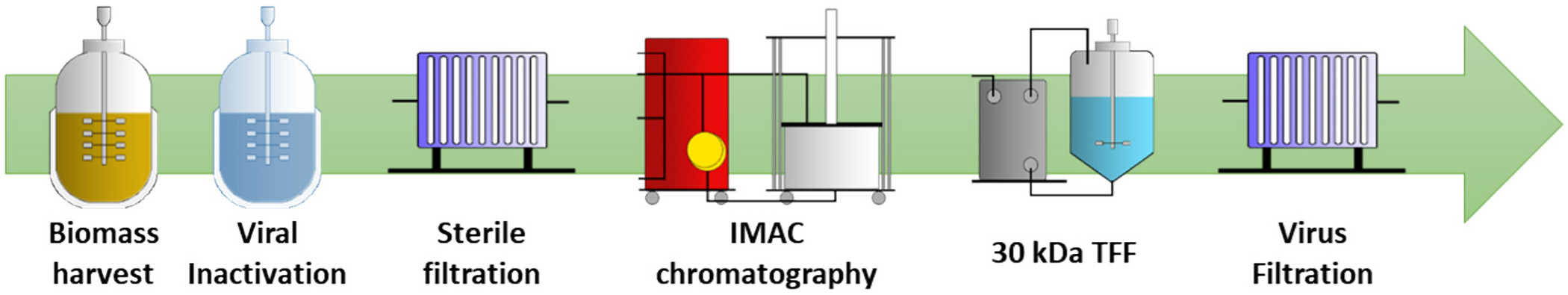
Process overview. After protein expression in insect cell culture, biomass is harvested, treated with 0.1 % Triton X-100 for virus inactivation, and sterile filtered. The clarified cell culture fluid is then loaded onto an IMAC column, and the eluate is buffer-exchanged using hollow fiber ultrafiltration.

While many studies in the literature outline methods for isolating and purifying NAs, comprehensive downstream strategies that meet good manufacturing practice (GMP) for pharmaceutical products are still lacking. Achieving these standards requires effective removal of process impurities, ensuring they remain below specific thresholds typically defined during process development in collaboration with regulatory agencies [19]. In this study, the development of a downstream process for purifying of His-tagged recombinant neuraminidase for a phase I clinical study is presented, incorporating detergent-based viral inactivation, immobilized metal affinity chromatography (IMAC), and tangential flow filtration (TFF) (Fig. 1).

While designing downstream processes, process-related limitations must be considered. For example, published research suggests that exposure to both Ni^2+^ and imidazole during purification can negatively impact NA enzymatic activity [20]. In this context, reducing loading times during chromatography may be desirable in order to prevent problems related to protein activity. Furthermore, ensuring reproducibility across batches is critical for maintaining consistency in purified material.

One of the primary impurities to monitor is DNA, with World Health Organization directives setting a limit of 10 ng per parenteral dose for vaccines [21]. Other important impurities include host cell proteins (HCP), which are product- and dose-specific, and typically defined during clinical trials. In the case of using immobilized metal affinity chromatography (IMAC), it is also important to account for the potential leakage of metal ligands such as nickel into the final product. Regulatory agencies advise minimizing heavy metal exposure, with tolerance limits typically set in the microgram-per-day range [22,23].

In addition, the removal of adventitious agents such as enveloped viruses is critical, and guidelines recommend including orthogonal methods to remove and/or inactivate those, such as viral filtration and incubation with specific detergents. This can be achieved through the use of nanomembranes featuring a retentive region with a pore size of 15–20 nm, and of detergents like Triton X-100, which disrupt viral envelopes [24,25]. Still, these detergents must also be effectively removed throughout the process. Typically, Triton X-100 is added early in the purification process and is subsequently eliminated during chromatography wash phases and buffer exchange steps, and a variety of analytical techniques employed to verify its removal [26,27].

The downstream process proposed here is designed with scalability and industrial compatibility in mind. It includes measures to monitor and control key parameters, such as residual enveloped viruses, Triton X-100 concentration, and potential nickel leakage from the resin. This streamlined approach removes impurities at each stage, with DNA, HCP levels, and other critical contaminants carefully tracked and kept under recommended limits. Finally, the purified product is thoroughly characterized to assess protein concentration, tetrameric integrity, stability, and enzymatic activity.

Studies defining immunological properties and integrity of the construct N1-MPP expressed in small scale using the baculovirus expression system were previously performed. [13–18] This work characterizes the purification process in larger scale and its impact on the purified protein, with the intent of defining further protein features in order to potentially structure purification platform for tag-less N1-MPP. The process described aims to generate N1-MPP to be tested for Phase I clinical trials.

## 2. Materials and methods

### 2.1. Generation of recombinant baculovirus expressing neuraminidase

The N1-MPP construct was developed using the baculovirus expression system in *Spodoptera frugiperda* Sf9 cells as previously described [28]. The N1-MPP gene sequence was cloned into a pFastBac vector and transformed into DH10Bac *E.coli* cells for recombinant bacmid generation as per manufacturer instructions. The N1-MPP gene sequence included an N-terminal signal peptide followed by a hexahistidine purification tag, a measles virus phosphoprotein (MPP) tetramerization domain, and the N1 globular head domain from A/Michigan/45/2015 (H1N1) virus. The recombinant bacmid encoding the N1-MPP gene was transfected into Sf9 cells cultured in ESF AF medium in a 6-well plate at 27 °C and recombinant baculovirus (rBV) harvested at day 5 post-transfection (P0) by centrifugation at 2000×*g* for 5 min. The P0 was sequentially amplified in Sf9 cells for two additional rounds in T-175 flasks. The material produced during these experiments was used for all the experiments described in Section 3.1.

The N1-MPP gene was cloned into the multi-cloning site of pvL1393 using standard cloning practices. The resulting pVL1393-N1-MPP plasmid was co-transfected with BestBac 2.0 Linearized Baculovirus DNA (Expression Systems, Davis, CA) in Sf9 cells cultured in ESF AF (Expression Systems, Davis, CA) as per manufacturer’s instructions. The co-transfection culture was incubated at 27 °C and harvested on day 5 post-transfection. The resulting P0 virus was purified by end point dilution to isolate a single virus clone expressing N1-MPP. The P1 clone 3A-D2 was amplified for three additional rounds to a P4 virus stock. The P4 virus stock was used for all subsequent expression cultures using Sf9 cells in ESF AF. The P3 ESF AF virus stock was amplified in Sf9 RV-Free cells cultured in ESF AdvanCD to a P4 stock for subsequent expression cultures using Sf9 RV-Free cells cultured in ESF AdvanCD. The material produced in these experiments was used during all the experiments reported in Section 3.2.

### 2.2. Expression in Sf9 cells cultured in ESF AF

Sf9 cells adapted for growth in ESF AF cell culture media (Expression Systems, Davis, CA) were seeded to a density of 2.5 × 10^6^ cells/ml and incubated shaking at 27 °C. Approximately 24 h post-seed, the culture was infected with the N1-MPP expressing baculovirus at a multiplicity of infection (MOI) of 3. Six hours post-infection, the culture was supplemented with 5 % Production Boost Additive (PBA). The culture was harvested 72 h post-infection. At the time of harvest, the cells were pelleted by centrifugation and the supernatant filtered through a 0.2 μm filter, and the clarified supernatant frozen at −80 °C. The upstream expression process was moved to Sf9 RV-Free cells in ESF AdvanCD (Expression Systems, Davis CA) chemically defined culture medium. This allowed the elimination of Sf-rhabdovirus from the process, thus removing a known source of concern in drug manufacturing process development [29]. Sf9 RV-Free cells in ESF AdvanCD were seeded to a density of 4.0 × 10^6^ cells/ml and incubated shaking at 27 °C. Approximately 24 h post-seed, the culture was infected with the N1-MPP expressing baculovirus at a multiplicity of infection (MOI) of 3. The culture was harvested 50 h post-infection. At the time of harvest, the cells were pelleted by centrifugation and the supernatant supplemented with Triton X-100 to a final concentration of 0.1 % v/v. In order to determine whether Triton X-100 concentration used for the viral inactivation step would impact subsequent purification steps efficiency, aliquots of the supernatant were collected and incubated with either 0 % or 1 % v/v Triton. The Triton X-100 treated supernatant was incubated at room temperature for 30 min with agitation before filtration through a 0.2 μm filter and freezing at −80 °C.

### 2.3. Downstream process

During process development experiments, clarified cell culture batches were incubated with either 0 or 0.1 % Triton X-100 for a time period ranging between 10 min and 1 h, with or without agitation. These changes, however, did not impact product recovery or process outcome. Hence, the experiments described in Section 3.1 were carried out using clarified cell culture fluid that was incubated with 0.1 % Triton X-100 for enveloped virus inactivation at 30 °C for 1 h. Afterwards, the supernatant was sterile filtered using a 0.22 μm polyethersulfone membrane and loaded onto Ni Sepharose Excel (Cytiva) resin volumes ranging between 1.6 and 2 mL, manually packed in a Tricorn 10–100 housing. The column was equilibrated for 10 column volumes (CVs) in equilibration buffer (phosphate buffer 20 mM pH 7.4, 500 mM NaCl), and volumes of supernatant ranging between 125 and 250 CVs were loaded. Afterwards, the column was washed for 10 CVs with equilibration buffer and for 30 CVs with washing buffer (phosphate buffer 20 mM pH 7.4, 500 mM NaCl, 30 mM Imidazole). Finally, the product was eluted in a 5 CVs linear gradient elution between washing buffer and elution buffer (phosphate buffer 20 mM pH 7.4, 500 mM NaCl, 500 mM Imidazole). The fractions containing neuraminidase were immediately collected and buffer exchanged in formulation buffer (20 mM Tris-Base, 100 mM NaCl, pH 7.5) using Amicon Ultra-15 30 kDa centrifugal filters. The centrifugal filters were initially washed with formulation buffer, then loaded with the fractions to be buffer exchanged. Centrifugation was carried out at 4000*g* for 5 min, and the process was repeated until a target volume was permeated.

During the scaled-up process characterization experiments described in Section 3.2, the clarified cell culture fluid was incubated with final concentrations of Triton X-100, ranging between 0 and 1 % v/v for enveloped virus inactivation at 30 °C for 1 h, followed by sterile filtration using a 0.22 μm polyethersulfone membrane. The supernatant was loaded onto 11.25 mL Ni Sepharose Excel (Cytiva) resin, manually packed in a HiScale 16 housing and connected to an ÄKTA Pure 25 system. IMAC capture was performed as described above. The fractions containing neuraminidase were collected and buffer exchanged in formulation buffer (20 mM Tris-Base, 100 mM NaCl, pH 7.5) using a 30 kDa MidiKros membrane connected to an ÄKTA Flux. Tangential flow filtration (TFF) was operated in a semi-continuous mode, where the level of the reservoir was replenished throughout the process until 5–6 diafiltration volumes were permeated. During all IMAC processes, the eluate from all steps, from the flow-through (FT) to the elution phase, was collected, pooled, and analyzed. Following the filtration step, the permeate and the retentate were collected and analyzed.

### 2.4. Total protein, DNA, and Sf9-HCP quantification

For determination of total protein content, Pierce BCA Protein Assay Kit (Thermo Fisher Scientific, Waltham, MA, USA) was used. The assay was performed in a 96-well plate according to the manufacturer’s instructions. The calibration curve was obtained by using Pierce Bovine Serum Albumin Standard Pre-Diluted Set (Thermo Fisher Scientific) with a concentration range from 25 to 1000 μg/mL. Double stranded DNA (dsDNA) was determined by Invitrogen Quant-iT PicoGreen dsDNA kit (Thermo Fisher Scientific) in a 96-well plate according to the manufacturer’s instructions. The calibration curve was obtained by diluting the standard in series with Tris-EDTA buffer (included in the kit) to a concentration ranging from 0.195 to 25 ng/mL.

To determine the amount of insect cell host cell protein (HCP) impurities in intermediate process fractions and the final product, the third-generation Sf9 HCP ELISA kit (Cygnus Technologies, Leland, NC, USA) was used according to manufacturer instructions. The calibration curve was obtained using the prediluted standard with a concentration range of 3–200 ng/mL.

### 2.5. Electrophoresis

Sodium dodecyl sulfate–polyacrylamide gel electrophoresis (SDS-PAGE) was performed in an X-cell SureLock Mini-Cell electrophoresis chamber (Invitrogen, Carlsbad, CA, USA), using NuPAGE Bis/Tris 4–12 % gels (Invitrogen, Carlsbad, CA, USA) and reduced (2-(N-morpholino) ethanesulfonic acid)-Sodium dodecyl sulfate (MES-SDS) running conditions at 200 V and 400 mA for 50 min. Samples were prepared with NuPAGE LDS sample buffer (Invitrogen, Carlsbad, CA, USA) and incubated at 95 °C for 15 min in the presence of 182 mM dithiothreitol (DTT). For each sample, a volume of 25 μL (10-well gels), 20 μL (12-well gels) or 15 μL (15-well gels) was loaded in a gel lane. SeeBlue Plus2 Prestained Protein Standard marker (Thermo Fisher Scientific) was used as molecular weight marker for SDS-PAGEs and Western blots. After electrophoretic separation, the gels were incubated in fixing solution containing 50 % ethanol, 10 % acetic acid for 20 min and stained for 30 min in the staining solution containing 0.1 % Coomassie R-250 in 40 % ethanol, 10 % acetic acid. The gels then were destained with a solution containing 25 % ethanol and 8 % acetic acid until the background was clear and the bands were clearly visible.

For Western blot analysis after SDS-PAGE, proteins were transferred from the gel to a 0.2 mm nitrocellulose membrane using the Trans-Blot Turbo Transfer System (Bio-Rad, Hercules, CA, USA) according to the manufacturer’s instructions. Membranes were blocked overnight with 3 % *w/v* BSA (A7030, Sigma Aldrich, St. Louis, MO, USA) in Phosphate Buffer Saline (PBS) with Tween 20 (0.1 % *w/v* Tween 20 in PBS). Detection was performed using a two-step procedure: at first, the membranes were incubated with 5 mL 1:2000 diluted 4A5 anti-N1 antibody against NA in PBS-T containing 1 % w/v bovine serum albumin (BSA) for two hours [10]. The second step was the incubation of the membranes with the anti-mouse IgG conjugated with alkaline phosphatase (Sigma Aldrich), diluted 1:1000 in PBS-T with 1 % w/v BSA for one hour. Premixed 5-bromo-4-chloro-3-indolyl-phosphate/nitro blue tetrazolium (Sigma Aldrich) was used as substrate for visualizing the alkaline phosphatase conjugates. Between each antibody incubation step and the final visualization step, the membranes were washed with PBS-T by gently agitating, using an orbital shaker. To determine the isoelectric point of N1-MPP, isoelectric focusing (IEF) was performed using the Invitrogen IEF Kit (Thermo Fisher Scientific). The Invitrogen Novex pH 3–10 IEF protein gels (Thermo Fisher Scientific) were filled with 7 μL of IEF marker or 10 μL of sample (diluted 1:1 in sample buffer) and separation was performed using constant 100 V for two hours with an expected current of 7 mA at the beginning and 5 mA and the end. The same staining solution as in the SDS-PAGE was used, fixing and destaining was done according to the manufacturer’s instructions.

### 2.6. Calorimetry

The melting point of N1-MPP was determined using differential scanning calorimetry (DSC) using Nano DSC (Waters, Milford, MA, USA). The loops were filled with degassed buffer or sample according to the manufacturer’s instructions and scans were performed from 25 °C to 95 °C with a rate of 1 °C/min. The melting point of N1-MPP was also confirmed through differential scanning fluorimetry (DSF) using the “Uncle DSF System” (Unchained Labs). Briefly, 9 μL of purified N1-MPP were pipetted as is in the dedicated sample cassette. Full range fluorescence scans were performed while the samples were heated from 25 to 95 °C, at the rate of 1 ° C/min. The 350/330 nm ratio over the temperature range was analyzed through the MoltenProt tool, allowing for curve fitting and melting temperature (Tm) extrapolation by using the first order derivative. [30,31]

### 2.7. Sandwich enzyme-linked immunosorbent assay

N1-MPP was quantified using a sandwich enzyme-linked immunosorbent assay (ELISA). Immulon 4 HBX 96 well plates (Thermo Fisher Scientific) were coated with 2 μg/mL 100 μL per well 1G01 capture antibody, diluted in PBS, covered and stored overnight in 4 °C [32]. The plate was washed 3 times with 250 μL PBS-T and blocked with 220 μL 3 % BSA (*w/v*) in PBS-T. After blocking and washing, sample or standard with a concentration range of 2.5 ng/L to 5 mg/L was added and the plates were incubated at RT for two hours. After washing, 100 μL of the biotinylated anti-NA antibody 2H08 diluted to 5 μg/mL in blocking solution were added [33]. After incubation for one hour at room temperature, the plates were washed and 100 μL of streptavidin-conjugated horseradish peroxidase (HRP, Thermo Fisher Scientific) diluted 1:3000 in blocking solution were added and incubated for one hour at room temperature. After washing the plates 4 times, 100 μL SigmaFast *O*-phenylenediamine dihydrochloride solution (Sigma-Aldrich) was added and the reaction stopped after 10 min with 50 μL 3 M HCl. The absorbance at 490 nm was measured immediately after adding the stopping solution.

### 2.8. Enzymatic activity measurements

To determine NA activity, NA-star influenza neuraminidase inhibitor resistance detection kit (Thermo Fisher Scientific) was used. 96-well solid white flat bottom polystyrene tissue culture-treated microplates (#3917 Corning, Corning, NY, USA) were filled with 100 μL of antigen, in serial dilution and incubated for 20 min at 37 °C. 10 μL of 1:1000 diluted NA-star substrate was pipetted in each well and further incubated for 20 min at RT. After 20 min, 60 μL of NA-star accelerator was pipetted in two rows and the luminescence was measured immediately. For ELISA and NA-star assay, signal was plotted against dilution and N1-MPP concentration per well respectively. The resulting data was evaluated by using the statistics software R and fitting a 4-parameter log-logistic model from the dose response curve (drc) package Values with a residual standard error of 20 % were used for evaluation [34]. The interpolation results were reproduced in Python, which was used also to visualize the data. [35]

### 2.9. Analytical chromatography

Reversed phase high pressure liquid chromatography (RP-HPLC) was carried as previously described [36]. A Waters separation module e2695 (Milford, USA) was employed for all experiments. The column used was the TSKgel Super-Octyl C8 column (2.3 μm, 4.6 × 100 mm, Tosoh Bioscience, Griesham, Germany). The temperatures of the column oven and the auto-sampler were set to 50 °C and 4 °C, respectively. A constant flow rate of 2 mL/min was selected, with the column being equilibrated in a 90:10 ratio of A (99.9 % HQ-water, 0.1 % trifluoric acid) to B (99.9 % acetonitrile, 0.1 % trifluoric acid). Following a 1 min and 30 s period of maintaining a composition of 90 % A and 10 % B, a gradient was initiated, increasing the B composition to 60 % over a period of 18 min and 30 s. Detection was performed using a UV/Vis detector in dual wavelength mode (214 and 280 nm), with a wavelength of 214 nm being employed for quantitative evaluation. The resulting chromatograms were analyzed using Empower 3 software. Once the first RP-HPLC analyses of purified N1-MPP samples proved to be reproducible, the method was validated for quantification purposes. To do so, samples for calibration experiments were prepared from a 2 g/L N1-MPP stock solution to achieve final concentrations of 0.94, 0.82, 0.52, 0.28, 0.16, 0.09 and 0.05 g/L. As the volume of sample injected onto the column for the experiments was 20 μL, the amount of N1-MPP applied onto the column ranged between 19 and 1 μg. Homoscedasticity was evaluated by F-test of the variances of low and high concentrations, and linearity was tested by ANOVA linearity test and lack-of-fit test. Precision and accuracy was determined by calculating relative standard deviation and bias of duplicate injections at varying concentrations on eight days, respectively.

High pressure size exclusion chromatography (HP-SEC) was also performed by employing the Waters separation module e2695 (Milford, USA). In short, purified N1-MPP was loaded onto a Superdex 200 Increase 10/300 GL column (GE Healthcare), equilibrated with 20 mM Tris and 100 mM NaCl at pH 7.5. The flow rate was set to 0.4 mL/min, and the column was operated at room temperature. Detection was performed at 280 nm using a UV/Vis detector. Data acquisition and analysis were conducted using Empower 3 software (Waters). The resulting chromatograms were examined to identify the presence of any peaks eluting before the 26-min mark, which would indicate the presence of aggregates.

### 2.10. Nickel quantification

Samples were digested in 1.5 mL perfluoroalkoxy vials on an acid-resistant hot plate (100 mg sample + 200 μL ultrapure HNO3 + 100 μL ultrapure H2O2; *n* = 3) at 90 °C for 70 min, spiked with 100 μL of an internal standard solution and filled up with ultrapure water to a final volume of 1.10 g. The sample digests were measured by Inductively coupled plasma sector field mass spectrometry (ICP-SFMS) (Element2). Isotopes monitored were ^60^Ni and ^115^In (internal standard). Quantification was performed by external calibration and internal standardization using certified ICP-MS standard solutions (ICP multi-element standard VI, ICP single element standard solution, Merck). Accuracy and stability of sample analysis was controlled by RM NWTM 23.5 lot 1122 (trace element matrix reference material from diluted Lake Ontario water) and CRM Seronorm Trace Elements Serum L-2 as well as QC-Standards prepared from a certified multi-element standard solution (ICP multi-element standard VI, Merck).

### 2.11. Baculovirus quantification

The infectious baculovirus titer was determined using a flow cytometric method that is based on detection of the gp64 baculovirus fusion protein on the surface of infected cells and has a limit of detection (LOD) of 1 × 10^5^ infectious units per milliliter (IU/mL) [37]. Briefly, Sf9 cells were plated in wells of a 96-well plate at a rate of 2 × 10^5^ cells per well. Serial 10-fold dilutions of the unknown virus, as well as a standard control virus of known titer, were prepared and 100 μL of the 10-fold serial dilutions were pipetted into wells containing cells and incubated overnight shaking at 155 rpm at 27 °C. 18–20 h post inoculation, the cells were pelleted by centrifugation and resuspended in a 1:200 dilution of phycoerythrin (PE) labeled anti-gp64 antibody, clone AcV1 (Expression Systems, Davis CA). The plates were incubated for 15 min at 4 °C and subsequently washed three times in PBS. Cells were analyzed by flow cytometry using the detector in the PE channel to determine the percentage of gp64 positive cells in each well. A standard curve was generated from the wells infected with serial dilutions of the control virus and the equation generated from the standard curve was used to calculate the titer of the unknown virus sample.

## 3. Results & discussion

### 3.1. Process development: Initial considerations

N1-MPP was expressed in Sf9 cells using the baculovirus expression vector system (BEVS), with the protein fused to a 6 × His-tag for efficient affinity chromatography capture. Sterile-filtered Sf9 cell culture supernatant was loaded onto IMAC resin, operating close to the manufacturer’s recommendations. Initial process development considerations accounted for the column volumes (CVs) loaded onto the resin, as well as the required residence time (t_R_) during this phase. Given the low initial protein titer, ranging between 9 and 12 mg of N1-MPP per liter of supernatant, it was crucial to maximize the loading volume to ensure sufficient target product concentration in the eluate.

**Fig. 2.**
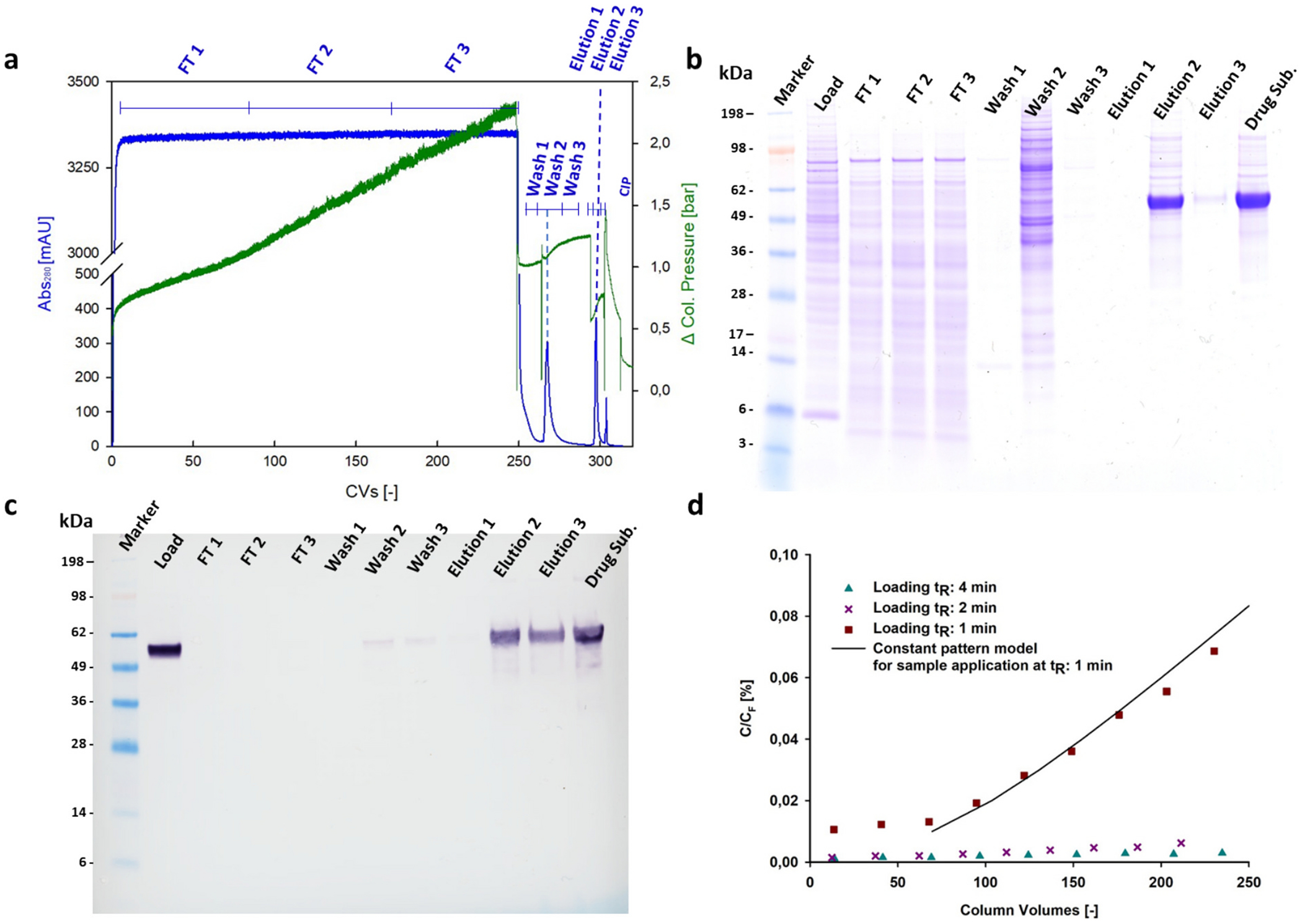
Overview of process development experimental results. Panel a: Chromatogram reporting absorbance (left axis) and pressure profile (right axis) in function of the elution volume. Process phases are highlighted and markers are set to indicate the pooled volumes collected. Panel b: SDS-PAGE showing protein content in the pooled fractions. A dominant band can be observed in the lane E2, corresponding to the peak observed during the elution phase. Drug substance refers to the protein concentration after buffer exchange performed by centrifugal ultrafiltration. Panel c: Western Blot assay performed using N1-MPP targeting antibodies. Panel d: amount of N1-MPP detected through ELISA in flow through (FT) fractions after loading the resin at different t_R_, compared to constant pattern solution model.

Preliminary experiments involved loading 250 CVs of clarified cell culture fluid onto a chromatographic column, setting 4 min t_R_, followed by protein elution over 5 CVs (Fig. 2a). During these experiments, a sharp gradient was preferred over a step elution phase in order to enhance process robustness across scales while not drastically affecting product yield or concentration. [19] SDS-PAGE analysis of the elution fractions showed a dominant band around 55 kDa, corresponding to the N1-MPP monomer (Fig. 2b). Western blot analysis with anti-N1-MPP antibodies confirmed that this band represented the desired product (Fig. 2c). No product was detected in the flow-through or in the first wash fraction, indicating effective binding during the loading phase. Trace amounts of N1-MPP were observed after washing with 30 mM imidazole buffer. Additionally, DNA content was measured (Table 1), confirming that this key impurity was reduced within acceptable limits after a single purification step.

Following this workflow, over 9 mg of N1-MPP per liter of cultivation were produced, with a 97 ± 4 % reduction in contaminating proteins and a 3.5 log reduction in DNA content. The absence of product loss during chromatography further encouraged continued process optimization.

However, the process required over 20 h to complete, and in some cases, the column pressure exceeded the critical pressure of 3 bar, potentially compromising resin integrity. In some instances, precipitate was observed in the flow through fractions, indicating low colloidal stability of the processed material. Also, to maintain product stability and minimize potential issues associated with prolonged exposure to Ni^2+^ ions—such as reduced enzymatic activity or potential protein oxidation—it was also desirable to minimize the loading time. Subsequent process development focused on evaluating the influence of t_R_ on the resin’s dynamic binding capacity. A set of experimental breakthrough curves was generated with clarified cell culture fluid at t_R_ of 1, 2, and 4 min, to visualize the relationship between N1-MPP breakthrough and flow rate (Fig. 2d). At higher flow rates and low residence times, breakthrough should increase because of mass transfer limitation. [19]

Due to the aforementioned loading constraints, experiments were halted after 250 CVs. In these cases, anti-N1-MPP ELISA and Western blot analyses revealed almost no product in the flow-through fractions when the column was loaded at 4 and 2 min t_R_, while up to 7 % breakthrough was observed at a 1 min t_R_ (Fig. 2d). This behavior was modeled using the constant pattern solution proposed by Weber and Chakraborti as previously described [38,39]. It should be noted that this model provides reliable estimates for parameters such as resin capacity and pore diffusivity, but requires full breakthrough curve data for optimal accuracy. Therefore, having only observed 7 % breakthrough of N1-MPP, model predictions only give an approximate prediction of true breakthrough behavior. If lower resin utilization is acceptable, loading 125 CV onto the column at a 1 min tR would enhance productivity and throughput, resulting in a fast and efficient affinity chromatographic step. This resolution yields a high-purity product at concentrations suitable for drug development and pharmacological use. The advantages of such a process setup would include high product recovery and a rapid chromatographic step, where large volumes of supernatant are loaded onto the resin in approximately 2 h. Buffer exchange and product concentration were achieved using ultrafiltration membranes with a 30 kDa molecular cut-off. Initial experiments performed using centrifugal ultrafiltration resulted in high product recovery of over 90 %. This process was scaled up to tangential flow filtration.

### 3.2. Process characterization

The optimized process was characterized in regards to total protein, DNA, and HCP (Table 2). SDS-PAGE and Western blot analyses confirmed the presence and identity of N1-MPP in the elution fractions. Mass balance calculations indicated that the majority of contaminants were effectively removed, with most flowing through the column or being washed away in the washing steps of the chromatographical process.

Therefore, extensive testing and repeated trials were conducted to ensure reproducibility and effectiveness, laying the foundation for subsequent validation. Specifically, five chromatographic runs, each followed by TFF buffer exchange and concentration, were performed. Throughout these steps, yield, impurity removal and N1-MPP concentration were monitored (Table 3).

**Fig. 3.**
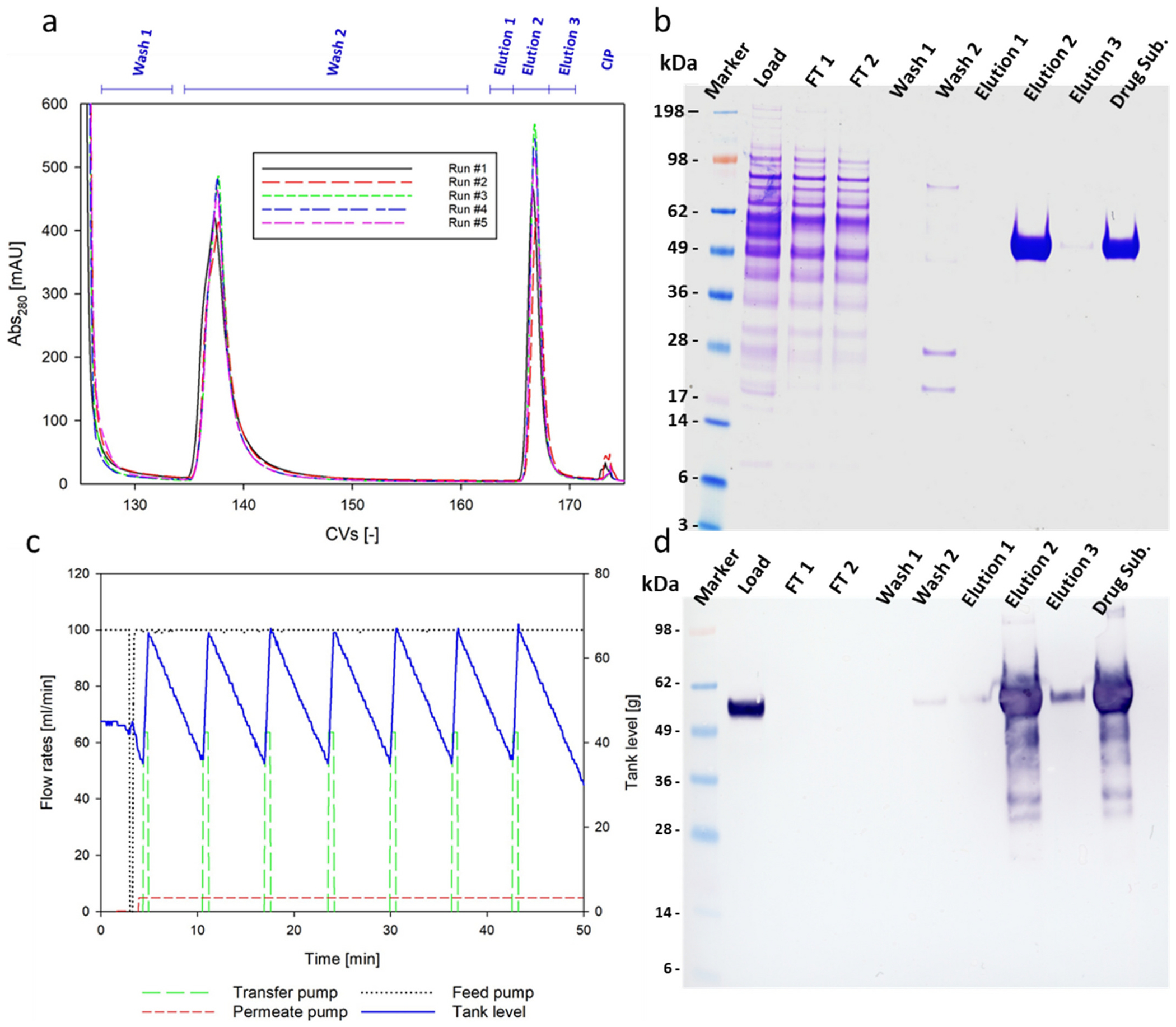
Overview of process characterization experimental results. Panel a: overlay of the absorbance signal detected during the chromatography characterization experiments in function of the elution volume expressed in CVs. Panel b: exemplary SDS-PAGE of the fractions collected during the experiments. Panel c: exemplary report of the TFF run data in function of experimental time (min): on the left axis, transfer pump, permeate pump, and feed pump flow rates are reported, while the amount of material present in the tank (grams) is stated on the right axis. Panel d: Western blot assay performed using N1-MPP targeting antibodies. The sample pattern is the same reported for the SDS-PAGE. It is worth noticing that samples were not diluted prior to being loaded on SDS-PAGE. Consequently, Elution and Drug Substance lanes appear overloaded.

Despite slight variations in initial process conditions, such as CVs loaded or the amount of Triton X-100 used to inactivate enveloped viruses, the output remained consistent across all runs. Overlaying absorbance signals as a function of elution volume revealed no significant differences between processes (Fig. 3a), a finding further supported by protein and DNA mass balances. DNA per dose never exceeded 10 ng, while HCP levels varied between 10,000 and 14,000 ppm. The main variable resulted to be final N1-MPP concentration detected in the drug substance, as maintaining a consistent final volume at the end of the TFF process (Fig. 3c) proved to be challenging in small scale. All TFF unit operations yielded over 90 % N1-MPP recovery in all runs, and 98 % imidazole was removed on average after permeating five diafiltration volumes. 40 % DNA removal was observed after buffer exchange, while HCP levels were not considerably impacted. SDS-PAGE (Fig. 3b), Western Blot (Fig. 3d) and ELISA confirmed N1-MPP purity and identity in the eluate fractions in all these experiments.

Reducing SDS-PAGE showed a band corresponding to the NA monomer at approximately 55 kDa in the elution peak in all characterization runs, constituting over 99 % of band intensity of the respective lane. Western blot analysis confirmed the presence of the target protein in both the load and elution fractions. Traces of the product were also detected in wash fractions in some runs. Notably, no traces of N1-MPP were detected in the flow through fractions by Western blot analysis.

Varying the Triton X-100 concentration used to inactivate enveloped viruses at the start of the process did not impact the final yield or purity. Triton X-100 concentrations in all final buffer-exchanged samples were below 1 ppm, and impurity levels remained consistent across all runs. A critical contaminant to monitor during process development is the amount of Ni^2+^ that leaches from the resin during IMAC. In this study, nickel content in the final samples was measured using ICP-SFMS and ranged between 25 and 40 μg/L, corresponding to an average of 3 ng per dose. No clear trend, such as diminishing leakage, was observed across the runs. Although some ligand loss occurred during repeated processes on the same resin, it did not significantly impact product recovery, likely the result of low resin utilization. Clearance of infectious baculovirus by the chromatography step was evaluated by purification of N1-MPP expression supernatant that had not been treated with Triton X-100. The infectious baculovirus titer of the column load, flowthrough, wash, and pooled eluate was determined and the resulting titers were 3.63 × 10^8^ IU/mL, 3.40 × 10^8^ IU/mL, 2.33 × 10^7^ IU/mL, and below the LOD respectively. This demonstrates that essentially all of the baculovirus passed through the column and was detected in the flowthrough and wash fractions. Given that the LOD of the baculovirus titering assay is 1 × 10^5^ IU/mL, the chromatography step alone results in a minimum three log reduction in infectious baculovirus titer. It is expected that the 0.1 Triton X-100 treatment will further reduce the baculovirus titer, and when the process is scaled up for GMP production, the nanofiltration process with reduce the baculovirus titer even further.

### 3.3. Product characterization

Amongst the analytical solutions employed to assess identity, purity and physicochemical properties of the purified N1-MPP drug substance, high pressure liquid chromatography was employed. Specifically, size exclusion chromatography was used to gain information in regards of tetramer integrity, while reversed phase chromatography offered further insights on sample purity and proved to be a valuable tool for N1-MPP and Triton X-100 detection and quantification.

**Fig. 4.**
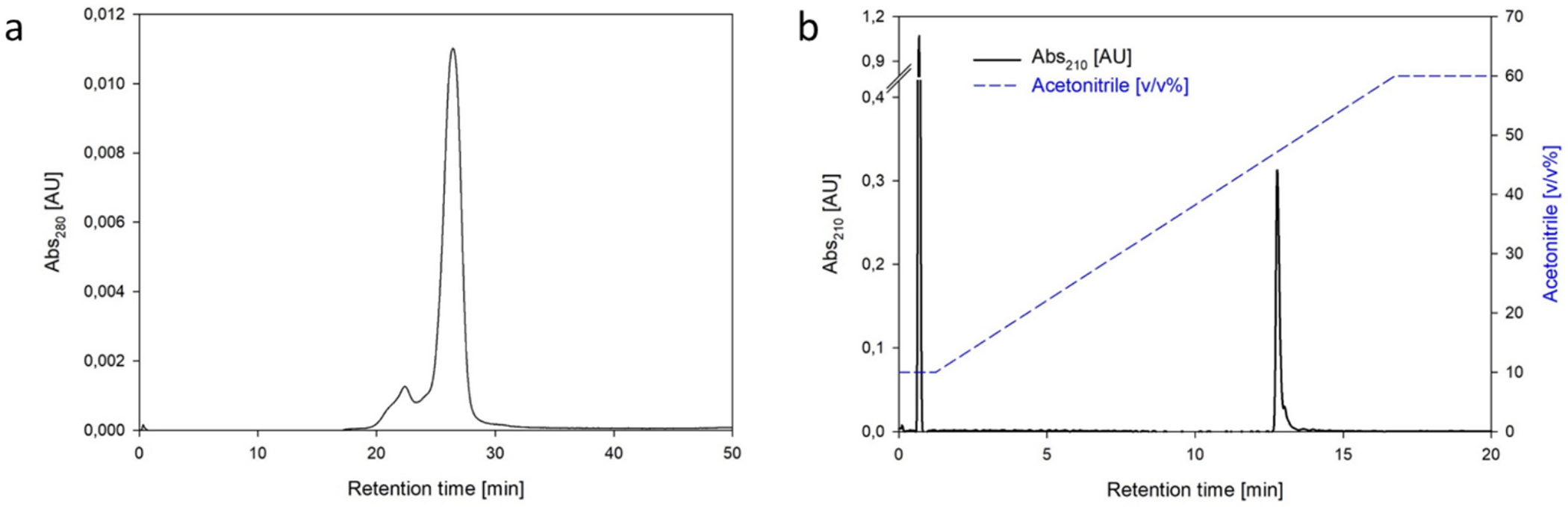
Analytical chromatograms of purified N1-MPP. Panel a: Exemplary size exclusion chromatogram observed for the purified drug substance. Panel b: Exemplary reversed phase chromatogram observed for the purified drug substance. On the left axis, the absorbance signal at 214 nm wavelength is reported, while on the right axis the acetonitrile concentration [*v*/v %] is shown.

Size exclusion chromatography (Fig. 4a) revealed a peak eluting at 26 min, corresponding to 0.43 CV. The injection of standard proteins of known molecular mass confirmed the correlation between this elution time and the expected molecular weight of the N1-MPP tetramer. These experiments showed no evidence of the presence of monomers or dimers in solution in any of the purified samples produced. Instead, high molecular weight (HMW) impurities, likely caused by partial aggregation, were also detected in the chromatogram. In N1-MPP purified samples, HMW impurities ranged from 10 % to 15 % of the chromatogram area. Other studies have reported the presence of higher order recombinant neuraminidase oligomers where the recombinant neuraminidase is expressed in combination with a His-tag [20]. In addition, size exclusion chromatography proved to be a valuable tool for quantifying residual imidazole in the purified N1-MPP fractions after buffer exchange, as this substance absorbs at 210 nm wavelength. The average residual imidazole concentration in purified N1-MPP produced during the experiments described in Section 3.2 (*n* = 5) was 4.6 mM.

RP-HPLC revealed a minor solvent peak followed by a single peak at approximately 47 % *v*/v acetonitrile concentration, accounting for over 99 % of the total chromatogram area. (Fig. 4b) The chromatogram profile was consistent across all analyzed final samples, further confirming both the efficiency of impurity removal and the reproducibility of the process.

A key advantage of this analytical method lies in its ability to detect Triton X-100. To determine whether increasing the amount of Triton X-100 used for enveloped virus inactivation would lead to detectable levels in the final purified product, the method’s sensitivity was evaluated, yielding a limit of detection (LOD) of 1 ppm for Triton X-100. Regardless of the amount of Triton X-100 introduced, no traces were detected in the final products, nor in the elution fractions indicating that this detergent was likely removed during the chromatography process.

**Fig. 5.**
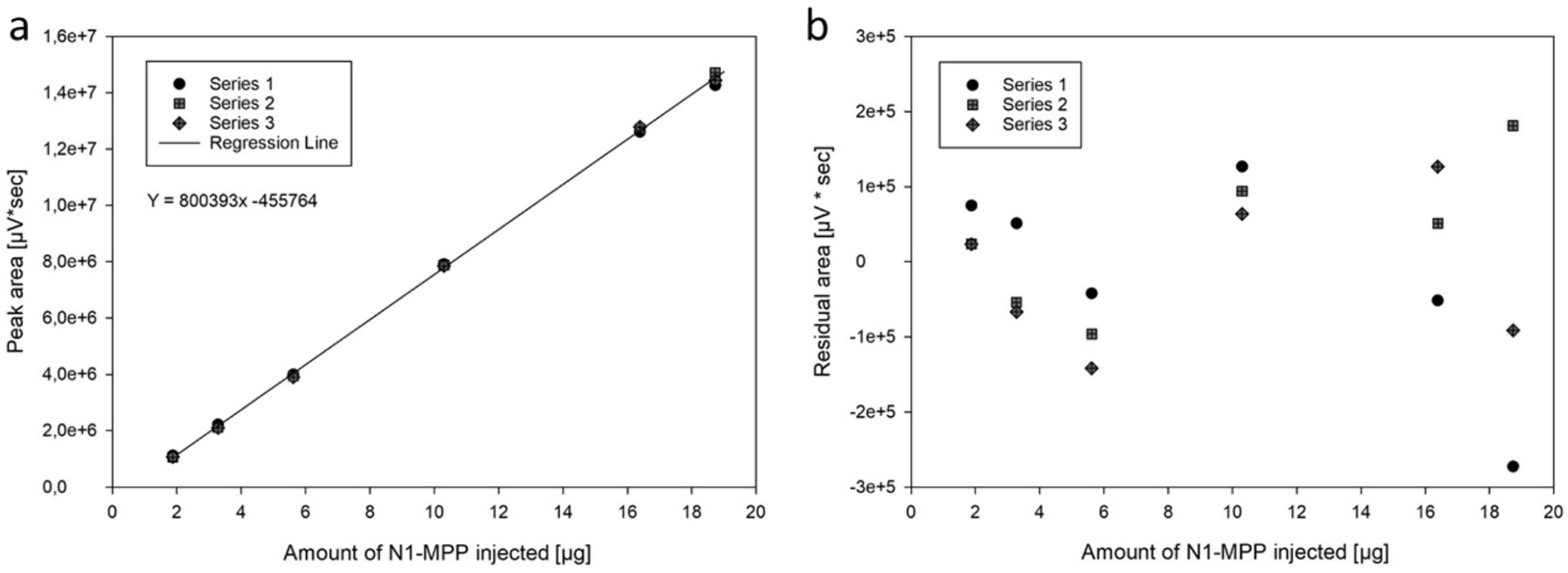
Validation of reversed phase HPLC method. Panel a: calibration line extrapolated after loading different amounts of purified N1-MPP on a TSKgel Super-Octyl C8 column. Panel b: residuals of the calibration line.

Interestingly, traces of N1-MPP could be detected and identified in the clarified cell culture fluid as well, suggesting that the N1-MPP tetramer could be analytically isolated on the basis of its hydrophobic properties. These results encouraged further efforts to validate this technique as a quantification tool for N1-MPP. In order to confirm the linearity of the calibration model and to identify the linear range of quantification, varying amounts between 0.94 and 18.73 μg of N1-MPP were injected in triplicates. Given the poor accuracy and precision values of 26.7 % and 24.6 %, respectively, 0.94 μg was excluded from the calibration curve and defined as the limit of detection. The next lowest standard, 1.87 μg, demonstrated acceptable accuracy and precision values for the RP-HPLC method (Table 4). The results of the injections of 1.9, 3.3, 5.6, 10.3, 16.4 and 18.7 μg are presented (Fig. 5 and Table 4). A linear response (Fig. 5a) was determined in the tested range, as evidenced by the results of the analysis of variance (ANOVA) linearity test (F* 35276.81 > F 8.53, α = 0.01), lack of fit test (F* 1.66 < 5.41 α = 0.01), and the absence of a discernible trend. The residuals were equally distributed (Fig. 5b).

**Fig. 6.**
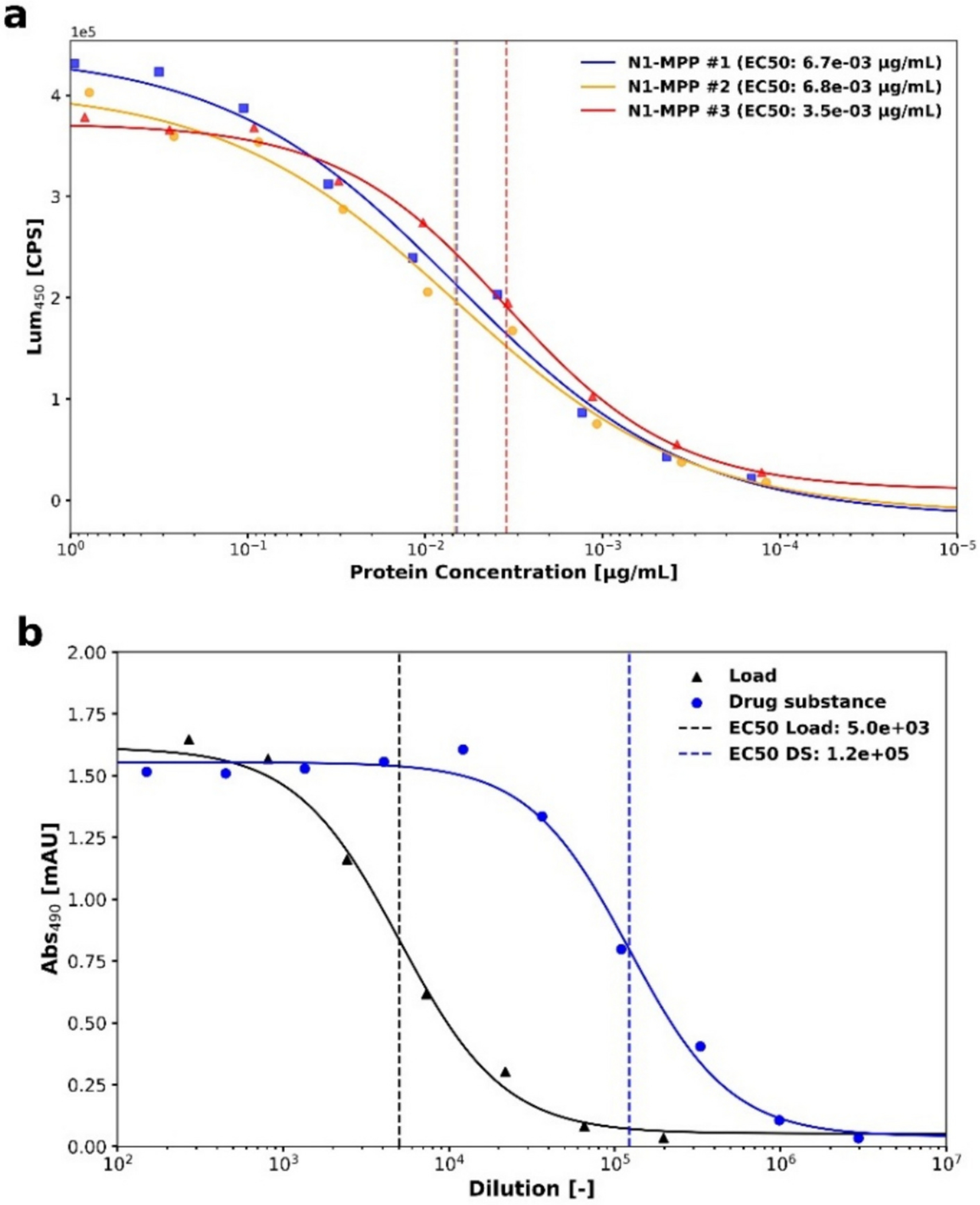
Panel a: 4PL fitted curves of exemplary samples produced during different process characterization experiments described in Section 3.2. EC_50_ values range between 3.5 e-03 and 6.8 e-03 as shown by the dashed line. Panel b: Exemplary results from N1-MPP ELISA on samples produced during process characterization experiments described in Section 3.2. Proportionality between EC_50_ values (outlined by dashed line) in the clarified cell culture fluid (Load, black curve, triangle marker, EC50: 5.0e+03) and purified sample (drug substance, blue curve, circular marker, EC50: 1.2e+05) can be seen. (For interpretation of the references to colour in this figure legend, the reader is referred to the web version of this article.)

The enzymatic assay results confirmed that the purified N1-MPP is active (Fig. 6a); however, the enzymatic activity did not scale proportionally with the concentration factor in different steps of the process. This suggests that while Na-MPP retains its functional integrity after purification, factors such as protein aggregation, partial misfolding, interfering enzymatic activity in the supernatant, or suboptimal assay conditions may be affecting its enzymatic efficiency at higher concentrations. Previous studies suggest that presence of histidine tags on recombinant neuraminidase may increase heterogeneity in its enzymatic activity. [20] While the results reported in this study show consistency with previously published data [13], it would be interesting to compare enzymatic activity amongst multiple histidine tagged and tag-less neuraminidase constructs produced in large scale. It is relevant to mention that multiple studies previously underlined the concept that “native conformation of NA is necessary for induction of NI antibodies; enzyme activity provides a useful marker of intact structure, but absence of activity in NA that is correctly folded does not result in loss of immunogenicity”. [40–43]

ELISA results demonstrated that NA-binding antibodies successfully bind to the purified N1-MPP protein (Fig. 6b), confirming the retention of key antigenic epitopes post-purification (Fig. 6b). This indicates that the structural integrity of the protein, is preserved throughout the purification process, making it suitable for serology and vaccine development. EC_50_ values for the purified N1-MPP samples correlated with protein concentration determined through BCA assay. Furthermore, as the described assay selectively binds native N1 NA epitopes, these results provide further confirmation of tetrameric integrity. [33]

**Fig. 7.**
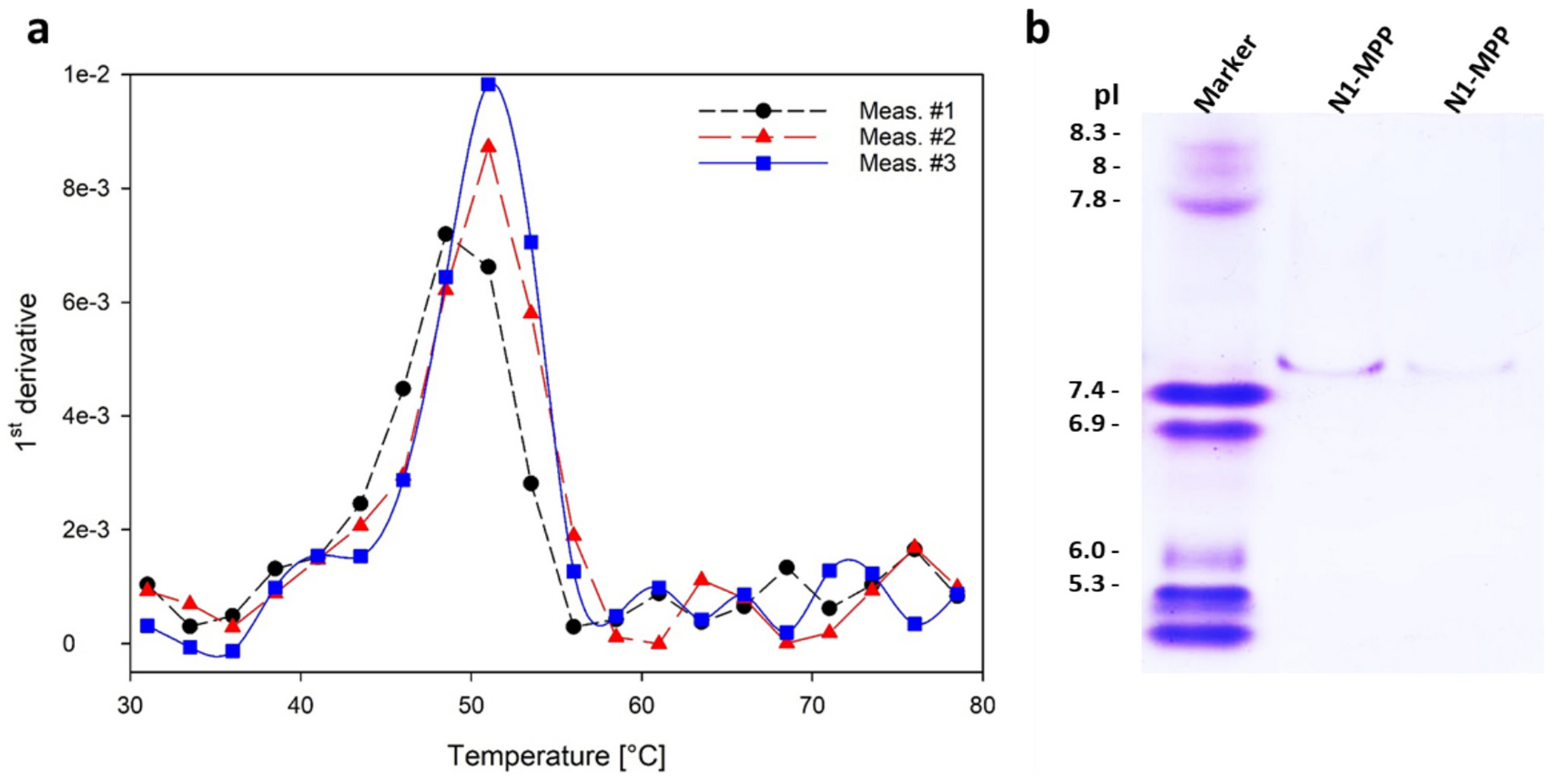
Panel a: First derivative of three DSF thermograms of purified N1-MPP. The graph shows one main peak centred around 49.9 ± 0.9 °C. Panel b: IEF of purified N1-MPP produced during two process characterization experiments described in Section 3.2. The samples, buffer exchanged and diluted, show a single migration band in the 7.4–7.5 range.

Purified N1-MPP was further analyzed to evaluate protein properties; the purified protein at the end of the process was collected and protein stability and isoelectric point were assessed through DSF and IEF gels. DSF analysis revealed a single thermal event at 49.9 ± 0.9 °C for the tetrameric protein, suggesting that the subunits unfold cooperatively and indicating strong inter-subunit interactions, as shown in Fig. 7a. This implies that the protein maintains a stable quaternary structure under the tested conditions, with no intermediate unfolding states detected. This can be observed in the first order derivative of the 350/330 nm wavelength trend in function of the increasing temperature (Fig. 7a).

IEF showed a pI of 7.4, (Fig. 7b) indicating the protein carries no net charge at physiological pH and may be less soluble and more prone to aggregation. This highlights the need for careful buffer selection during purification and formulation to maintain stability, especially under physiological conditions. Additionally, this information lays the basis for the development and optimization of purification conditions in techniques such as ion-exchange chromatography [44]. Nevertheless, the His-tag might also impact the isoelectric point of the protein, which may vary if the tag itself would be removed.

Overall, the downstream process developed for N1-MPP purification demonstrated a highly efficient and reproducible workflow, balancing purity, yield and process scalability between 0.5 and 100 L of supernatant. The integration of viral inactivation steps, IMAC and TFF consistently delivered a high purity product. DNA impurities were reduced to an average of 5 ng per dose, and never exceeded 10 ng across all purifications, while yielding more than 10 mg of purified N1-MPP per liter of cell culture. Minimizing the loading time during the chromatographic steps contributed to preserving resin integrity throughout the characterization experiments, during which no issues with pressure in the column were observed. Several techniques, such as SDS-PAGE, Western blot, ELISA and enzymatic assays confirmed N1-MPP identity. Additionally, DSF revealed a single thermal event at 49.9 °C, indicative of a structurally stable tetramer, and IEF gels confirmed a pI of 7.4. Further functional assays, including NA-star and ELISA, confirmed both enzymatic activity and antigenicity, establishing that the purified neuraminidase retained is active and displays a native conformation. The high yields obtained using this process support the scalability of this purification strategy, making it an attractive option for vaccine development.

## 4. Conclusion

We successfully developed and characterized a scalable, GMP-ready downstream process to produce a N1-MPP-based vaccine candidate. The optimized process achieved the required purity and potency for the drug substance in under seven hours of process time using only one chromatography step. The process demonstrated robustness across different upstream batches, ensuring consistent yield and quality. The purified material enabled detailed product characterization, informing the design of future purification platforms for tag-less neuraminidase. This work lays the foundation for further structural and functional studies of NA and supports the development of novel influenza vaccines with the potential for broad protection.

## Figures and Tables

**Table 1:** Exemplary total protein and DNA mass balance throughout preliminary chromatographic process where 450 mL of clarified cell culture fluid were loaded on IMAC resin manually packed in a 10–100 Tricorn housing. UF = ultrafiltration.

Preliminary run - protein and DNA mass balance - IMAC operation						
Sample	Conc.	Vol.	Protein Amount	Yield	dsDNA Amount	LRV
	[μg/mL]	[CV]	[mg]	[mg/L]	[ng]	[−]
**Load**	8973	248.6	4038	8973.3	1,296,475	0.0
**FT**	8747	248.6	3936	8746.7	1,277,767	0.0
**W1**	606	15.0	16	36.5	18,767	1.8
**W2**	344	12.2	8	16.8	2001	2.8
**W3**	<LOD	17.8	–	–	441	3.5
**E1**	<LOD	1.7	–	–	37	4.5
**E2**	686	4.4	5.5	12.2	674	3.3
**E3**	<LOD	1.7	–	–	75	4.2
**Preliminary run - protein & DNA mass balance - 30 kDa centrifugal UF E2 Load on**						
**UF**	686	3.6	4.46	9.9	548	3.4
**Drug Sub.**	944	2.5	4.2	9.4	403	3.5

**Table 2:** Exemplary mass balance of total protein, DNA and HCP at the end of IMAC and TFF operation.

Mass Balance: IMAC								
Sample	Volume	Volume	Total Protein	Recovery	Total DNA	Recovery	Total HCP	Depletion
	[mL]	[CV]	[mg]	[%]	[ng]	[%]	[ng]	[%]
**Load**	1410.0	125.3	5264.1	100.0 %	2.3E+05	100.00 %	9.5E+07	100.00 %
**FT pooled**	1410.0	125.3	5052.0	96.0 %	2.1E+05	91.85 %	8.1E+07	85.43 %
**W1**	112.5	10.0	48.0	0.9 %	9.8E+03	4.33 %	3.9E+06	4.14 %
**W2**	250.0	22.2	11.5	0.2 %	3.0E+03	1.33 %		
**E1**	8.0	0.7	<LOQ	<LOQ	4.9E+01	0.02 %	ND	ND
**E2**	40.0	3.6	15.0	0.3 %	3.5E+03	1.53 %	2.6E+05	0.28 %
**E3**	32.0	2.8	<LOQ	<LOQ	1.4E+02	0.06 %	ND	ND
**Mass Balance: TFF - Hollow Fiber**															
**E2 Load on TFF**	38.0	3.4	14.3	–	3.3E+03	–	2.6E+05	–
**Drug Substance**	32.2	2.9	16.8	0.3 %	1.9E+03	0.84 %	2.3E+05	0.24 %
**TFF Permeate**	214.0	19.0	<LOQ	<LOQ	<LOQ	<LOQ	ND	ND

**Table 3:** Overview of five purification runs, showing the loading volume, Triton X-100 concentration used during virus inactivation, process yield, and impurity levels measured as DNA (ng/dose) and host cell protein (HCP, ppm).

Parameter	Units	Process 1	Process 2	Process 3	Process 4	Process 5
**CV Loaded**	[−]	125	110	125	125	125
**[Triton X-100]**	[v/v%]	0.1	0.1	0.1	0	1
**Yield (Protein/L cultivation)**	[mg/L]	12	10	12	12	10
**Final concentration**	[μg/mL]	366	284	522	456	377
**DNA per dose**	[ng]	4.2	8.5	5.1	8.8	5.7
**HCP per dose**	[ppm]	10,670	10,598	13,604	11,071	14,087
**Residual Ni Content**	[μg/L]	36.7	39.8	33.3	38.1	24.8
**Residual Imidazole content**	[mM]	2.3	1.4	8.3	4.7	6.1

**Table 4:** Accuracy and precision of N1-MPP quantification method determined on eight days in duplicates (*n* = 16).

Standard [μg]	Accuracy	Precision	Note
0.9	26.7 %	24.6 %	Limit of detection
1.9	−0.8 %	12.4 %	Lower limit of quantification
5.6	−1.3 %	4.4 %	–
18.7	−0.9 %	2.7 %	Upper limit of quantification

## Data Availability

Data will be made available on request.
